# Prognostic assessment in patients operated for brain metastasis from systemic tumors

**DOI:** 10.1002/cam4.5928

**Published:** 2023-04-11

**Authors:** Bettina Grossenbacher, Anna Lareida, Selina Moors, Patrick Roth, Zsolt Kulcsar, Luca Regli, Emilie Le Rhun, Michael Weller, Fabian Wolpert

**Affiliations:** ^1^ Department of Neurology, Clinical Neuroscience Center University Hospital of Zurich, University of Zurich Zurich Switzerland; ^2^ Department of Neuroradiology, Clinical Neuroscience Center University Hospital of Zurich, University of Zurich Zurich Switzerland; ^3^ Department of Neurosurgery University Hospital of Zurich, University of Zurich Zurich Switzerland

**Keywords:** assessment, GPA, prognosis, score, surgery

## Abstract

**Background:**

Established models for prognostic assessment in patients with brain metastasis do not stratify for prior surgery. Here we tested the prognostic accuracy of the Graded Prognostic Assessment (GPA) score model in patients operated for BM and explored further prognostic factors.

**Methods:**

We included 285 patients operated for brain metastasis at the University Hospital Zurich in the analysis. Information on patient characteristics, imaging, staging, peri‐ and postoperative complications and survival were extracted from the files and integrated into a multivariate Cox hazard model.

**Results:**

The GPA score showed an association with outcome. We further identified residual tumor after surgery (*p* = 0.007, hazard ratio (HR) 1.6, 95% confidence interval (CI) 1.1–2.3) steroid use (*p* = 0.021, HR 1.7, 95% CI 1.1–2.6) and number of extracranial metastasis sites (*p* = 0.009, HR 1.4, 95% CI 1.1–1.6) at the time of surgery as independent prognostic factors. A trend was observed for postoperative infection of the subarachnoid space (*p* = 0.102, HR 3.5, 95% CI 0.8–15.7).

**Conclusions:**

We confirm the prognostic capacity of the GPA score in a cohort of operated patients with brain metastasis. However, extent of resection and steroid use provide additional aid for the prognostic assessment in these patients.

## Graphical abstract

Here we tested the prognostic accuracy of the Graded Prognostic Assessment (GPA) score model in patients operated for BM and explored further prognostic factors. We confirm the prognostic value of the GPA score in a cohort of operated patients with brain metastasis. However, extent of resection and steroid use provide additional aid for the prognostic assessment in these patients.

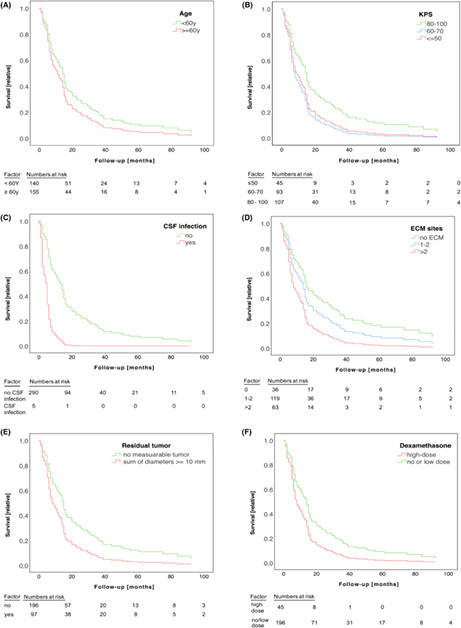

## 1 INTRODUCTION

Brain metastases (BM) from systemic cancer are the most common intracranial tumors.^1^, ^2^ Advances in multidisciplinary therapeutic concepts have improved outcome, from a median overall survival (OS) of about 4 months more than 20 years ago^3^ to more than 30 months in selected subgroups of contemporary large patient cohorts.^4^ Besides radiotherapy, chemotherapy, targeted therapies, and immunotherapy, neurosurgery has been established decades ago as an option for local treatment in patients with symptomatic BM or oligometastasis and a favorable overall condition.^5^, ^6^, ^7^, ^8^, ^9^ Improvement of surgical techniques by development of intraoperative MRI and electrophysiological mapping has reduced surgery‐associated morbidity and mortality.^10^, ^11^, ^12^, ^13^, ^14^ Prognostic assessment of patients operated for BM, however, remains difficult. Tools for estimation of prognosis such as the recursive partitioning analysis^3^ or the Graded Prognostic Assessment (GPA) score model^15^, ^16^, ^17^, ^18^, ^19^ were validated in cohorts with data from large radiation oncology trials. These models did not take into account whether patients were operated or not and it is unclear whether these predictive models are also valid for estimation of prognosis in BM patients after surgery.^14^ The goal of this investigation was to validate the GPA score in operated BM patients. Furthermore, we sought to identify clinical surgery‐related factors which are potentially associated with outcome. Such data could improve prognostic assessment in these patients after neurosurgery.

## 2 MATERIALS AND METHODS

### 2.1 Patients

Adult patients operated for BM from solid extra‐central nervous system tumors between January 2004 and December 2014 at the University Hospital Zurich were found by interrogation of the electronic documentation system. Survival was assessed from the time of BM detection by MRI imaging. At the timepoint of analysis, 235 of 285 patients (82%) had died, median follow‐up of the surviving patients was 40 months (95% confidence interval [CI] 32–48 months). The Cantonal Ethics Committee Zurich granted approval to this study (KEK‐ZH‐No. 2018‐00192). Consent was obtained according to local regulations for all patients included in this study.

### 2.2 Assessments

Pre‐operative data including clinical and imaging information were collected for the time frame of 4 weeks prior to brain surgery. Post‐operative assessment was performed during 4 weeks after the first surgery for BM, and included postoperative imaging, steroid dose, and clinical status. The highest documented dose of dexamethasone or equivalent dose of another steroid within 4 weeks prior to or after surgery was counted for assessment of pre‐ or postoperative steroid medication, respectively. Staging data from computed tomograms of chest and abdomen (CT) or combined with^18^ F‐fluorodeoxyglucose positron emission tomography (FDG‐PET/CT), histology of primaries and tumor‐specific treatment were collected. Information on the surgeries was extracted from the medical reports, including length of surgery. Postoperative complications such as cerebrospinal fluid (CSF) leak or CSF infection were extracted from the medical files, too.

Baseline and postoperative MRI imaging including T1‐sequences without and with contrast enhancement as well as T2 or FLAIR and blood sensitive MRI sequences such as susceptibility‐weighted images or gradient echo sequences were reviewed. Measurements included determination of sum of the largest diameters of target lesions according to Response Assessment in Neuro‐Oncology (RANO) recommendations.^20^ The formula: volume = ((length×width×height)/2)^21^ was used for volumetric assessment of intracranial BM. Bone metastasis was not calculated as part of the intracranial tumor volume. Oligometastasis was defined as 1–4 BM.

### 2.3 Statistical methods

Multivariate testing of variables potentially associated with outcome after surgery of BM was performed using a Cox hazard regression model. Potential confounding variables which have been previously identified were evenly integrated into the model. GraphPad Prism software, version 7.0 (La Jolla) and IBM SPSS statistics®, Version 23 (IBM Co., Armonk) were used for statistical evaluation. For two‐sided *p*‐values, results with *p* < 0.05 were considered significant and with *p* < 0.01 highly significant. Bonferroni correction for multiple testing was applied where appropriate.

## 3 RESULTS

### 3.1 Patient characteristics at the time of BM detection and after surgery

Database screening resulted in the identification of 295 patients with detailed information on patient characteristics including concurrent medical conditions, staging, neurosurgical procedure as well as pre‐ and postoperative imaging. Ten patients were excluded from further evaluation because pre‐operative scans were not available for review. Table 1 shows characteristics of the remaining 285 patients.

**TABLE 1:** Patient characteristics.

All patients (*n* = 285)	
Characteristics at detection of BM	
Sex, m/f (%)	149/136 (52/48)
Age in years, median (range)	62.2 (29.9–86.6)
Number of BM, median (range)	1 (1–12)
Oligometastasis (1–4 BM), *n* (%)	
No	23 (8)
Yes	262 (92)
KPS, median (range)	80 (40–100)
Dexamethasone dose within 4 weeks of detection in mg, mean (range)	11 (0–64)
Sum of longest diameters in mm, median (range)	34.7 (0–98.9)
Cumulative intracranial tumor volume in cm^3^, median (range)	11.6 (0.2–160.0)
GPA score at the time of BM detection, median (range)	2 (0–4)
Maximal radial diameter of edema of largest BM in mm, median (range)	25.5 (0–76)
Initial symptom or sign to perform MRI, *n* (%)	
No specific symptoms	25 (9)
First epileptic seizure	39 (14)
Headache	21 (7)
Nausea	4 (1)
Neurological deficit	42 (15)
Cognitive/personality change	13 (5)
Altered conscious state	3 (1)
Multiple	111 (39)
Other	25 (9)
No information	2
Symptoms from BM, *n* (%)	
No	25 (9)
Yes	258 (91)
Incomplete file	2
Localization of largest BM *n* (%)	
Deep brain	13 (5)
Cerebellum	53 (19)
Brain stem	12 (4)
Frontal	75 (26)
Parietal	56 (20)
Occipital	41 (14)
Temporal	28 (10)
Other	7
Extracranial metastasis, *n* (%)	
0	34 (16)
1–2 extracranial lesions	116 (55)
>2 extracranial lesions	60 (29)
Incomplete file	72
Largest BM necrotic, *n* (%)	
No	176 (64)
Yes	98 (36)
Not evaluable	11
Largest BM cystic (>1 cm), *n* (%)	
No	238 (86)
Yes	39 (14)
Not evaluable	8
Largest BM hemorrhagic, *n* (%)	
No	190 (73)
Yes	69 (27)
Not evaluable	26
Characteristics after first resection of BM	
Primary tumor according to BM histology, *n* (%)	
Unknown	7 (3)
Non small cell lung carcinoma	103 (36)
Small cell lung carcinoma	15 (5)
Melanoma	50 (18)
Breast cancer	29 (10)
Renal cell cancer	11 (4)
Gastrointestinal cancer	38 (13)
Other	32 (11)
Sum of longest diameters in mm, median (range)	0 (0–60.5)
Cumulative intracranial tumor volume in cm^3^, median (range)	0.07 (0–34.5)
Dexamethasone dose in mg, mean (range)	3 (0–16)
Dexamethasone change compared to presurgery status, *n* (%)	
Decreased	158 (73)
Stable	38 (18)
Increased	19 (9)
No information	70
Duration of preceding surgery in minutes, median (range)	190 (15–470)
Extent of resection and residual tumor volume, *n* (%)	
Gross total resection (no contrast enhancing lesion)	136 (48)
Subtotal resection (<5% contrast enhancing tumor)	77 (27)
Incomplete resection (5 ≤ 20% contrast enhancing tumor)	43 (15)
Partial resection (21 ≤ 90% contrast enhancing tumor)	19 (7)
Biopsy (≥90% contrast enhancing tumor)	10 (4)
Postoperative CSF leakage, *n* (%)	
No	246 (88)
Yes	35 (12)
Not evaluable	4
Postoperative CSF infection, *n* (%)	
No	280 (98)
Yes	5 (2)
Evolution of preoperative deficits after surgery, *n* (%)	
Never experienced deficits	19 (7)
Persistent deficits	46 (16)
Partially resolved deficits	132 (46)
Completely resolved deficits	61 (21)
New deficits	27 (10)
No information	6
Postoperative hemorrhage, *n* (%)	
No signs of hemorrhage	102 (37)
Intracavity hemorrhage	49 (18)
Cortical hemosiderosis	3 (1)
Subdural hematoma	14 (5)
Epidural hematoma	4 (1)
Hemorrhagic transformation of residual tumor	93 (33)
Multiple of these	14 (5)
Incomplete file	6

*Note*: The first part of the table shows patient characteristics at the time of detection of BM whereas the second part summarizes data at the first post‐operative control within 4 weeks after surgery. The left column shows the items, the right column shows respective values as indicated.

Abbreviations: BM, brain metastasis; CSF, cerebrospinal fluid; KPS, Karnofsky performance status.

The most frequent initial singular symptoms were deficits during neurological examination (42 of 283 patients, 15%), followed by epileptic seizures (39 of 283 patients, 14%). Most patients showed several symptoms and signs of disease at detection of BM (111 of 283 patients, 39%). Median duration of surgery was 190 min (range 15–470), the most frequent histology of primary tumor was non‐small cell lung cancer (115 of 281 patients, 41%), followed by melanoma (48 of 281 patients, 17%), gastrointestinal cancer (36 of 281 patients, 13%), other tumors (34 of 281 patients, 12%), and breast cancer (28 of 281 patients, 10%) (Table 1).

We report a median number of one BM per patient, a cumulative intracranial tumor mass of 11.6 cm^3^, sum of longest diameters of 34.7 mm and dexamethasone equivalent dose of 16 mg, along with a median maximal radial diameter of edema of 25.5 mm and a median GPA score of 2 as the most important findings.

Altogether, these baseline data are indicative of a high intracranial tumor load prior to surgery. Staging showed that the majority of patients had extracranial metastasis in one or more regions of the body, one third of patients in more than two sites (Table S1). Necrosis of the largest BM was found in approximately a third, hemorrhagic transformation in a fourth of patients prior to surgery. A cystic configuration of BM was found in 39 of 277 patients (14%).

A gross total resection was performed in 136 of 285 patients (48%) and a subtotal resection in 77 of 285 patients (27%) (Table 1). We also observed a significant decrease of steroid doses after surgery (median of the highest documented dexamethasone dose within 4 weeks after the detection of BM 16 mg/d vs. 0 mg/d within 4 weeks after surgery).

During the first 4 weeks of postoperative follow‐up, CSF leakage was documented in 35 of 281 patients (12%), CSF infection as confirmed by lumbar puncture was documented in 5 of 285 patients (2%). Clinical outcome after surgery showed new deficits in 27 of 279 patients (10%), the majority of patients showed partial or complete resolution of pre‐operative deficits according to the discharge reports. Postoperative imaging showed hemorrhagic transformation of residual tumor in 93 of 279 patients (33%) and bleeding into the resection cavity in 49 of 279 patients (18%); no hemorrhage was found in 102 of 279 patients (37%). Median OS of our cohort was 14 months (95%CI 11.9–16.1). There was no significant difference between the time period from 2004 to 2009 (OS 12 months, 95% CI 8.3–15.7) compared to the subsequent time period between 2010 and 2014 (OS 14 months, 95% CI 11.5–16.5, *p* = 0.56, log rank test). Postsurgical mortality within 30 days was 4% (13 of 295 patients).

### 3.2 GPA in patients operated for BM

**FIGURE 1:**
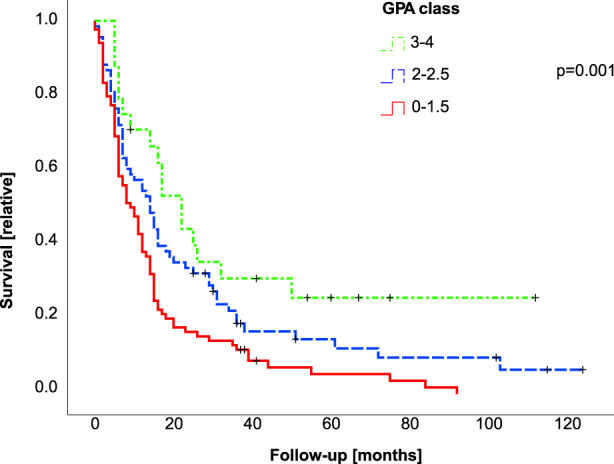
GPA score classes are associated with survival in operated patients with BM. The Kaplan–Meier survival curve shows outcome of patients with different GPA categories. The GPA score was calculated based on patient characteristics at the time of diagnosis. According to score results, patients were assigned to three different GPA categories with a score result of 0–1.5 (red straight line), 2–2.5 (blue dashed line), or 3–4 (green dot‐dashed line). *y*‐axis marks percentage of surviving patients and *x*‐axis survival in months. The log‐rank test was employed for comparison of groups, *p*‐values between groups are shown in brackets.

Information on age, extracranial metastatic lesions, number of BM and Karnofsky performance status (KPS) were extracted from the files and used for calculation of the score items.^22^ We report a median GPA score of 2 and assigned patients to three GPA categories: class I (GPA 0–1.5), class II (GPA 2–2.5), and class III (GPA 3–4). Kaplan–Meier analysis showed significant differences in survival between GPA groups (class I: OS 22 months, 95% CI 9.5–34.5; class II: OS 14 months, 95% CI 9.9–18.1; class III: OS 9 months, 95% CI 6.0–12.0; see Figure 1 for *p*‐values; Figure 1, Table 2), indicating that the GPA score is valid in the subgroup of patients operated for BM.

**TABLE 2:** Multivariate analysis on items of the GPA score for overall survival^a^.

Candidate factors	*p*	Odds ratio	95% CI	
			Lower	Upper
Age (<50 years vs. 50–60 years vs. 60 years)	0.027	1.4	1.1	1.8
Karnofsky performance status (80–100^b^, 60–70, ≤50)	0.003	1.4	1.1	1.7
Number of BM (1^b^, 2–3, ≥4)	0.005	1.3	1.1	1.6
Extracranial metastasis (no, yes)	0.361	1.1	0.9	1.5

*Note*: The table demonstrates multivariate Cox hazard analysis of items of the GPA score. Column one shows items of the score, column two two‐sided *p*‐values, column three odds ratios, the last two columns state 95% CI.

^a^ The results of multivariate testing by Cox regression analysis of items of the GPA score^15^ are shown. The first column depicts the respective candidate factor, the second one the two‐sided *p*‐values, the third column the respective odds ratios following 95% CI in the fourth and fifth column.

^b^ Indicates reference values for the respective analysis.

For calculation of the RPA score, another established model for prognostic assessment, we applied KPS, age, presence of extracranial tumor lesions and control of the primary tumor according to Response Evaluation Criteria in Solid Tumors (RECIST) 1.1 criteria.^23^ The majority of patients was assigned to RPA class II (*n* = 198 of 229 patients, 86%, Figure S1), followed by RPA class I (*n* = 19 of 229 patients, *n* = 8%) and RPA class III (*n* = 12 of 229 patients, 5.2%). The low number of patients with RPA class I and III limited survival analysis. Still, patients from the RPA Class I group showed a more favorable prognosis than patients from the RPA class II group (Class I: *n* = 19, median OS 22 months, 95% CI 2.9–41 vs. Class II: *n* = 198, OS 13 months, 95% CI 10.6–15.4 months; *p* = 0.02, log‐rank test). There was no difference between patients of the RPA Class III and other classes, although the low number of patients in this group was a limitation (Class III *n* = 12 patients, median OS 14 months, 95% CI 0–36 months; *p* = 0.053 vs. Class I and *p* = 0.162 vs. Class II).

**FIGURE 2:**
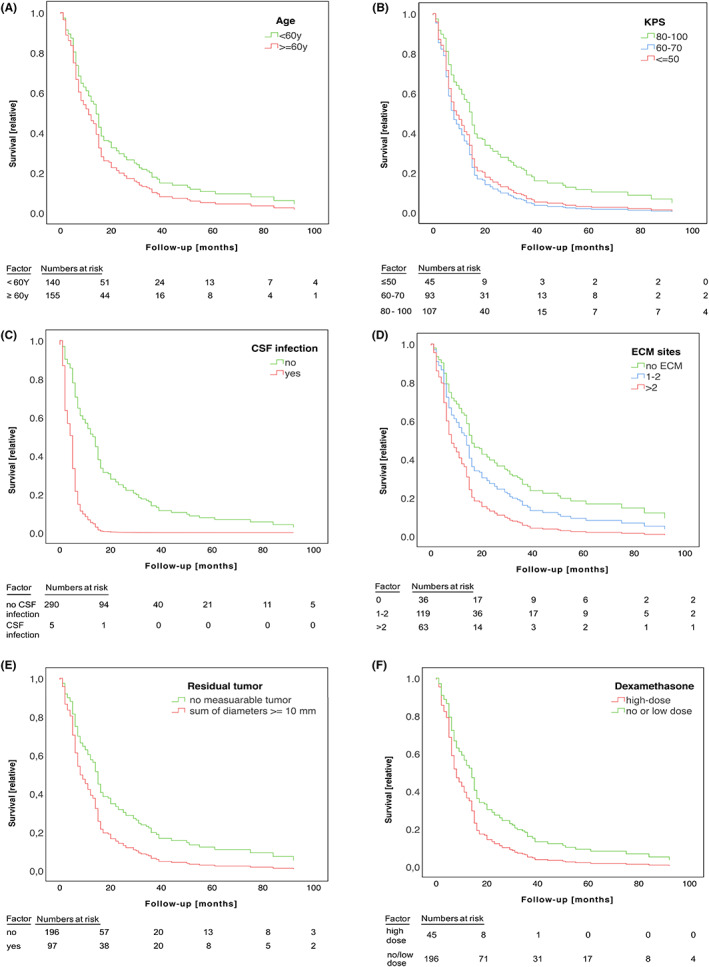
Multivariate assessment of possible surgery‐associated prognostic factors in BM patients. (A–E). Cox hazard curves are shown for candidates for prognostic factors. The percentage of surviving patients is shown on the y‐axis, the *x*‐axis marks follow‐up in months. A. Survival is shown for individuals of 60 years of age or above (red line) and patients younger than 60 years (green line), in (B) for individuals with a KPS of 80%–100% (green line), 60%–70% (blue line), and 50% or lower (red line), in (C) survival is shown for patients with (red line) or without (green line) CSF infection. In (D), survival is shown for patients with no (green line), one, or two (blue line) or more than two (red line) extracranial metastatic sites. (E) Survival data for patients with gross total tumor resection (green line) or measurable residual tumor (red line), (F) shows outcome of patients with higher dose dexamethasone (≥4 mg within 4 weeks after surgery, red line) compared to no or lower dose dexamethasone <4 mg, green line).

However, several risk factors which are specifically associated with surgery might have a significant influence on survival, but are not represented by the GPA model in its present form. We therefore computed a multivariate Cox regression analysis including several candidates for pre‐ and postoperative prognostic markers. Results of multivariate testing are shown in Figure 2, Table 3. Altogether, we found an independent association of KPS (Figure 2B, Table 3), extracranial metastasis burden (Figure 2D, Table 3), post‐operative sum of longest diameters as correlate of residual intracranial tumor volume (Figure 2E, Table 3) and higher dexamethasone dose (≥4 mg/d) following surgery (Figure 2F, Table 3). There is ongoing discussion if and how to correct for multiple testing in Cox regression analysis. The Bonferroni method is widely accepted as a standard for correction of multiple testing, but is regarded as possibly too conservative for application to the results of Cox regression analysis and might result in underestimation of effects.^24^ Using the Bonferroni method, the corrected significance level would have been adjusted from 0.05 to *p* = 0.009. If applying this adjusted, very conservative significance level, the association of dexamethasone and outcome would be rated as formally not significant, but would be still retained for KPS, number of extracranial metastasis sites and residual sum of longest diameters after surgery. We found a trend toward inferior survival for patients with CSF infection (Figure 2C, Table 3); however, this complication was rare (five of 285 patients). We found no association of outcome with sex (*p* = 0.302, hazard ratio [HR] 0.8, 95% CI 0.6–1.2), initial symptoms from BM (*p* = 0.280, HR 1.5, 95% CI 0.7–2.9), oligometastasis (*p* = 0.380, HR 0.8, 95% CI 0.5–1.3), or pathology of the primary tumor (*p* = 0.341, HR 1.1, 95% CI 0.9–1.2).

**TABLE 3:** Multivariate analysis of prognostic factors for survival after neurosurgical BM resection^a^.

Candidate factors	*p*	Hazard ratio	95% CI	
			Lower	Upper
Age (<60 years, > 60 years)	0.095	1.3	1.0	1.9
Karnofsky performance status (80–100, 60–70, ≤50)	0.008	1.4	1.1	1.8
CSF infection (no, yes)	0.102	3.5	0.8	15.7
Number of extracranial metastasis sites (0, 1–2, >2)	0.008	1.4	1.1	1.9
Residual sum of longest diameters after surgery (no measurable tumor, sum of longest diameters ≥10 mm)	0.007	1.6	1.1	2.3
Dexamethasone dose (<4 mg/d, ≥4 mg/d)	0.021	1.7	1.1	2.6

*Note*: Multivariate Cox hazard analysis of putative prognostic factors after neurosurgery is demonstrated as a table. The first row shows candidate factors, the subsequent rows two‐sided *p*‐values, odds ratios and 95% CI.

^a^ The results of multivariate testing by Cox regression analysis of prognostic factor candidates are shown. The first column depicts the respective candidate factor, the second one the two‐sided *p*‐values, the third column the respective odds ratios following 95% CI in the fourth and fifth column.

## 4 DISCUSSION

A survival benefit from surgery in subgroups of BM patients has been reported by several randomized controlled clinical trials decades ago.^5^, ^6^, ^7^ Several models for prognostic assessment have been developed and continuously improved in the last 25 years,^19^ however, only few recent investigations specifically focus on the subgroup of operated patients.^25^, ^26^, ^27^, ^28^ Lacking sufficient data, development of standardized criteria for prognostic assessment in these patients remains challenging.

According to current guidelines,^9^ the detection of single or symptomatic BM are indications for surgical resection, in particular if rapid relief from symptoms from increased intracranial pressure can be provided. Surgery is further recommended if there is uncertainty about the nature of a lesion, the primary tumor or the molecular signature. If a targetable mutation of the primary tumor cannot be confirmed in BM, targeted agents are unlikely to be effective.^9^

Here we provide robust data from a well‐defined large cohort of patients operated for BM. The established GPA score proved to be in general valid for prognostic assessment in the subgroup of operated patients (Figure 1), which is in line with a previous report.^27^ The uneven distribution of patients in our cohort indicate that RPA class might be not an optimal score for prognostic assessment in the subgroup of patients which are operated for BM due to bias from patient selection. Multivariate analysis of GPA score items, however, confirmed an association only for age, KPS and number of BM, but not presence of extracranial metastasis (Table 2). Refined prognostic assessment showed an association of outcome and the number of extracranial tumor sites, indicating that detailed staging information on the extracranial tumor load should be considered in the prognostic assessment. Furthermore, we identified postoperative residual tumor volume after operation of BM as independent prognostic factor. Another possible association was observed for ongoing higher dose medication with dexamethasone. This notion is supported by a previous report which states an association of steroid medication with inferior outcome in glioblastoma patients.^29^ Another report, however, did not show an association of grade of resection and outcome,^30^ this should be further addressed by subsequent controlled studies.

An association of higher age and CSF infection with outcome was only noticed as a trend (age: *p* = 0.095; CSF infection: *p* = 0.102, Figure 2, Table 3). This indicates that the association of higher age with outcome is possibly overruled by other prognostic factors. CSF infection was a rare finding in our cohort, and therefore this part of analysis might have been underpowered to finally test an association with outcome.

The retrospective character is the major limitation of our study from a single primary center, and the results should be confirmed in the course of prospective clinical trials. Furthermore, patients were included in the time frame from 2004 to 2014, the lack of more recent data represents another limitation.

In conclusion, we report here new prognostic factors in order to facilitate estimation of prognosis of patients after neurosurgical BM resection, including KPS, number of extracranial tumor sites, residual tumor and dexamethasone treatment. In the pre‐operative assessment, the feasibility of gross total resection and the extent of extracranial tumor load should be considered in order to identify patients likely to derive long‐term benefit from neurosurgical interventions.

## AUTHOR CONTRIBUTIONS

**Bettina Grossenbacher:** Conceptualization (equal); data curation (lead); formal analysis (equal); investigation (equal); visualization (supporting); writing – original draft (equal); writing – review and editing (equal). **Anna Lareida:** Data curation (equal); formal analysis (equal); investigation (equal); writing – review and editing (supporting). **Selina Moors:** Data curation (equal); formal analysis (supporting); investigation (equal); writing – review and editing (supporting). **Patrick Roth:** Formal analysis (equal); methodology (equal); resources (equal); writing – original draft (supporting). **Zsolt Kulcsar:** Formal analysis (supporting); resources (equal); validation (equal); writing – original draft (supporting). **Luca Regli:** Formal analysis (supporting); investigation (equal); resources (equal); writing – original draft (supporting); writing – review and editing (equal). **Emilie Le Rhun:** Formal analysis (equal); investigation (supporting); writing – original draft (supporting); writing – review and editing (equal). **Michael Weller:** Conceptualization (equal); formal analysis (equal); methodology (equal); project administration (equal); resources (lead); supervision (equal); validation (equal); writing – original draft (lead); writing – review and editing (lead). **Fabian Wolpert:** Conceptualization (lead); data curation (lead); formal analysis (lead); investigation (lead); software (equal); supervision (lead); writing – original draft (lead); writing – review and editing (lead).

## FUNDING INFORMATION

None.

## ETHICS STATEMENT

The Cantonal Ethics Committee Zurich granted approval to this study (KEK‐ZH‐No. 2018‐00192).

## Supporting information


**Figure S1:** RPA classes and survival in operated patients with BM. The Kaplan–Meier survival curves show outcome of patients with different RPA classes (RPA class I: blue curve; RPA class II: red curve, RPA class III: green curve). RPA classes were calculated based on patient characteristics at the time of diagnosis. *y*‐axis marks percentage of surviving patients and *x*‐axis survival in months. The Log‐Rank test was employed for comparison of groups, *p*‐values between groups are indicated in the legend.

**Table S1.** **Detailed staging information.** In the first column, the respective site of extracranial lesion are stated. The subsequent column shows values of patients as indicated.

## Data Availability

Upon reasonable request, original data from this study can be provided by the corresponding author.
