# Supplementary figures and images for: Prognostic assessment in patients operated for brain metastasis from systemic tumors
Source: Cancer Med. 2023 Apr 11;12(11):12316–24. doi: 10.1002/cam4.5928 (PMC10278502; doi:10.1002/cam4.5928)

**Figure S1**

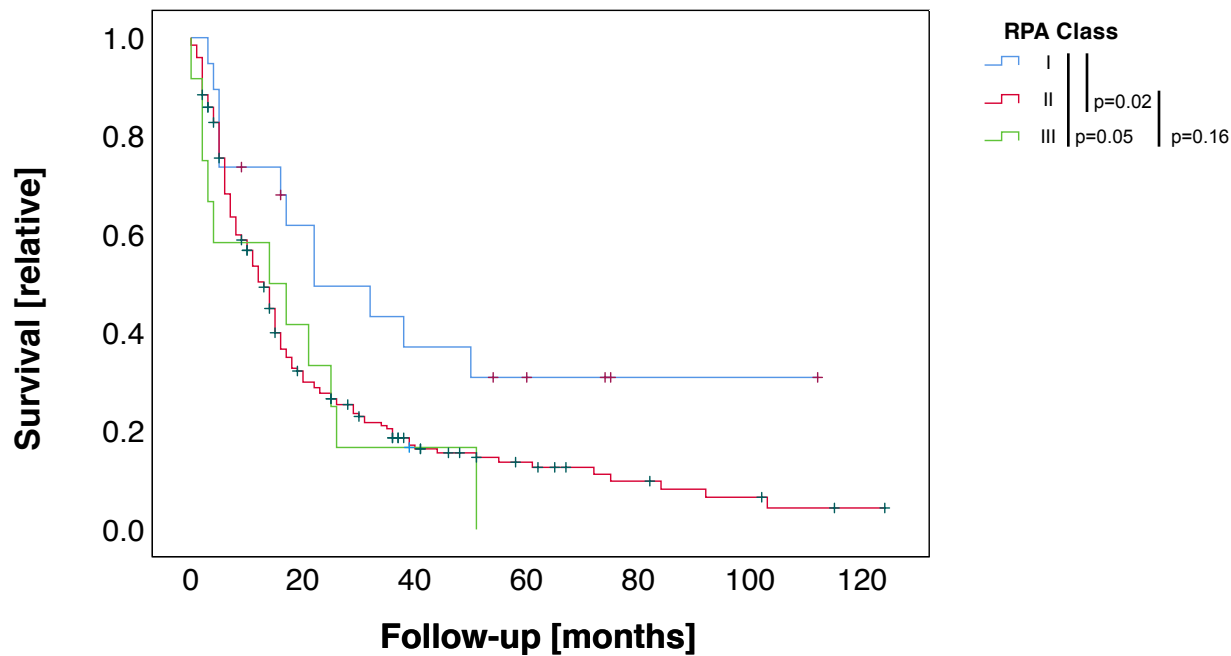

Supplement: Supplementary file 1 — Figure S1: RPA classes and survival in operated patients with BM. The Kaplan–Meier survival curves show outcome of patients with different RPA classes (RPA class I: blue curve; RPA class II: red curve, RPA class III: green curve). RPA classes were calculated based on patient characteristics at the time of diagnosis. y‐axis marks percentage of surviving patients and x‐axis survival in months. The Log‐Rank test was employed for comparison of groups, p‐values between groups are indicated in the legend. [file CAM4-12-12316-s002.pdf]
