# Supplementary material for: Prognostic assessment in patients operated for brain metastasis from systemic tumors
Source: Cancer Med. 2023 Apr 11;12(11):12316–24. doi: 10.1002/cam4.5928 (PMC10278502; doi:10.1002/cam4.5928)
Supplement: Supplementary file 2 — Table S1. Detailed staging information. In the first column, the respective site of extracranial lesion are stated. The subsequent column shows values of patients as indicated. [file CAM4-12-12316-s001.docx]

**Table S1. Detailed staging information**

|  | **All patients  (n=281)** |
| --- | --- |
| **Bone metastasis, n (%)** |  |
| no | 179 (83) |
| yes | 36 (17) |
| no information | 66 |
| **Liver metastasis, n (%)** |  |
| no | 186 (86) |
| yes | 29 (14) |
| No information | 66 |
| **Lung or pleural metastasis, n (%)** |  |
| no | 71 (33) |
| yes | 147 (52) |
| no information | 63 |
| **Mediastinal metastasis, n (%)** |  |
| no | 188 (87) |
| yes | 27 (13) |
| no information | 66 |
| **(Sub-)Cutaneous metastasis, n (%)** |  |
| no | 198 (92) |
| yes | 16 (8) |
| no information | 67 |
| **Kidney metastasis, n (%)** |  |
| no | 201 (72) |
| yes | 13 (5) |
| no information | 67 |
| **Lymph node metastasis, n (%)** |  |
| no | 116 (55) |
| yes | 97 (35) |
| no information | 68 |
| **Treatments administered after surgery of BM, n (%)** |  |
| no chemo- or radiotherapy | 24 (8) |
| radiotherapy only | 117 (40) |
| chemotherapy only | 7 (2) |
| chemotherapy and radiotherapy | 151 (49) |
| incomplete file | 3 |

**^+^** The results of database screening are shown. The first column depicts the respective characteristics item. The second column shows overall values for all patients, values as indicated.
